# Catalyst effect of human body odours in social anxiety treatment – a pilot study.

**DOI:** 10.1192/j.eurpsy.2024.48

**Published:** 2024-08-27

**Authors:** E. Vigna, V. Carli, C. Cecchetto, E. Dal Bò and C. Gentili from the Department of General Psychology, University of Padova, Padova, Italy. E. Eliasson, G. Hadlaczky and D. Wasserman from the National Centre for Suicide Research and Prevention of Mental Ill-Health, Karolinska

**Affiliations:** ^1^National Centre for Suicide Research and Prevention of Mental Ill-Health, Karolinska Institutet, Solna, Sweden

## Abstract

**Abstract:**

POTION is an EU funded project (No. 824153) within the Horizon2020 initiative that aims at understanding the nature of chemosignals in humans and their sphere of influence on social interaction. The emotional state of one person can be transmitted to another through volatile molecules contained, for example, in the sweat. These molecules, or chemosignals, are processed by the receiver who is not only able to identify the feelings of the sender but also to respond accordingly.

Whitin this project, we conducted a study with the aim of exploring the possible catalyst effects of body odour on social anxiety. We hypothesized that subjects exposed to human chemosignals, while undergoing mindfulness treatment, would show an enhanced reduction in anxiety symptoms in comparison to the control group (exposed to clean air).

To this aim, a study including 96 women aged between 18 and 35 years with symptoms of social anxiety was conducted. Ater recruitment, subjects were randomly allocated to one exposure group (happiness, fear or neutral human body odour or clean air) and followed a mindfulness intervention while being exposed to one of the odour or clean air. The same intervention was repeated twice, over two consecutive days. The main outcome was change in the State-Trait Anxiety Inventory (STAI) scores for which data was collected before and after treatment at each day. Mixed model analysis revealed significant changes in STAI scores in all groups during both days of trial. However, a greater decrease in anxiety symptoms was observed in subjects exposed to fear chemosignals during both days. A post-hoc comparison of the group exposed to clean air and the group exposed to fear chemosignals showed a trend level time x odour interaction during the second day of trial (F(1,45)=3.74, p=0.07).

In conclusion, our pilot study indicated a potential use of human body odours as a catalysts of social anxiety treatment. While the small sample size restricts the generalizability of our findings, the observed trends offer a promising foundation for future research.

**Disclosure of Interest:**

None Declared

